# Development of a convolutional neural network for the endoscopic classification of pouchitis in patients after restorative proctocolectomy

**DOI:** 10.1007/s10151-025-03273-6

**Published:** 2026-03-17

**Authors:** M. Saifi, U. Eisenmann, F. Ringwald, R. Liu, P. Kienle, D. Schmitz

**Affiliations:** 1 Department of General and Visceral Surgery, Brüderklinikum Julia Lanz – Diako Mannheim, Speyerer Straße 91-93, 68163 Mannheim, Germany; 2 Institute of Medical Informatics, Heidelberg University, Heidelberg, Germany; 3 Department of Gastroenterology, Helios Kliniken Schwerin, Schwerin, Germany

**Keywords:** Convolutional neural networks, Pouchitis, Inflammatory bowel disease, Endoscopy

## Abstract

**Background and aims:**

The aim of this prospective single-center study is to train convolutional neural networks (CNNs) to detect the presence of pouchitis in two-dimensional (2D) images acquired during pouchoscopies and test its feasibility.

**Methods:**

Two separate networks were constructed. The goal of network 1 was to detect whether an inflammation was present. Network 2 was designed to classify endoscopic findings of pouchitis, according to the pouchitis disease activity index (PDAI) score. The dataset was divided into three distinct sets: a training set, a validation set, and a test set. The performance was quantified using a tenfold cross-validation approach.

**Results:**

For the detection of inflammation, sensitivity was 71.78% with a specificity of 90.35%. When differentiating the six endoscopic findings according to the PDAI score, the sensitivity ranged from a low of 38% for the ‘ulceration’ class to a high of 67.18% for the ‘friability’ class, with a specifity of 94.12% (‘ulceration’) and 96.57% (‘friability’).

**Conclusions:**

This study shows that an artificial, intelligence-based image recognition software can be trained to recognize the endoscopic features of pouchitis with reasonable accuracy. The results, although encouraging, confirm that artificial intelligence (AI) performance in this context remains below human expert level. A larger dataset, human benchmarking and more appropriate endoscopic markers are required to reach clinically relevant performance.

*Trial registration* This trial was registered in the ‘ClinicalTrials.gov’ database on 26 April 2021 (NCT04864587).

## Introduction

Proctocolectomy with ileal pouch–anal anastomosis (IPAA) is the gold standard of surgical treatment for ulcerative colitis (UC) patients that require surgery, with pouchitis being the most common late complication. Pouchitis is characterized by a non-specific inflammation of the pouch. The reported incidence of pouchitis varies widely in the literature, with rates reaching up to 50% of patients within 10 years of the pouch procedure in large reference centre series [1, 2]. Pouchitis can present in both acute and chronic forms, with symptoms ranging from abdominal cramping, fever and increased stool frequency to perianal bleeding, abdominal discomfort, incontinence and nocturnal urgency. The pathogenesis of pouchitis remains unclear [3].

Genetic predisposition, such as variations in the NOD2/CARD15-Gen [4, 5], IL-1 receptor antagonist and Toll-like receptor genes [6, 7], as well as factors such as smoking cessation, previous severe UC episodes, backwash ileitis and extraintestinal manifestations, particularly primary sclerosing cholangitis (PSC), contribute to the risk of developing pouchitis [8–10]. In addition, regular use of non-steroidal anti-inflammatory drugs and the presence of other autoimmune diseases increase susceptibility [11–13]. Therapeutic options include antibiotics, probiotics and biologics [14–17].

Pouchoscopy is an essential tool in the diagnosis of pouchitis. Various diagnostic indices, in particular the pouchitis disease activity index (PDAI), are used to assess the severity of pouchitis [18]. However, the lack of international consensus on the classification of pouchitis poses a challenge in standardizing diagnostic criteria. Given the complexity of pouchitis diagnosis and management, there is an urgent need for innovative approaches to improve diagnostic accuracy. Leveraging advances in artificial intelligence (AI), particularly deep learning, offers promising opportunities to improve diagnostic accuracy and clinical decision-making in pouchitis. By developing a novel image recognition program based on the endoscopic features of pouchitis according to the PDAI score, we aim to investigate the potential of computer-aided diagnostics to assist clinicians in the assessment of pouchitis. This study addresses two key questions:

1. Is it possible to train a neural network to reliably detect an inflamed pouch (network 1)?
2. Is it possible to train a neural network for reliable scoring of the endoscopic features of the PDAI (network 2)?

## Material and methods

### Study design and ethics

The study protocol was reviewed and approved by the Ethics Committee of the Ruprechts-Karls-University Heidelberg (file no. 2021-571). Written informed consent was obtained from all participants.

The prospective single centre study is based on pouchoscopy videos, which were recorded during routine pouchoscopies using an ‘Evis Exera III’ gastrointestinal videoscope (Olympus K.K., Tokyo, Japan). The images that were utilized for the preliminary test (network 1) were sourced from different endoscopic systems. The indications for performing the examinations were made independently. These indications included annual check-up pouchoscopies (routine), pouchoscopies for acute symptoms of pouchitis and pouchoscopies to monitor success after treatment in the context of pouchitis. The videos of pouchoscopies used for the study are secondary data, which means that the data were collected exclusively as part of routine clinical care. The pouchoscopy videos were split into individual frames and assessed using a specially developed labelling software.

### Patients

The study included patients with colitis ulcerosa (CU) who were 18 years and older and who had undergone restorative proctocolectomy and ileoanal pouch creation at least 12 months before inclusion, starting with the reversal of the protective ileostomy.

### Gathering data

To minimize inter-examiner-dependent variances, an in-house standard operating procedure (SOP) was created, and all examining physicians were trained accordingly.

### Image assessment

The assessment of the images was carried out using the dual control principle. It was carried out by two raters with specialist qualifications as a minimum requirement. The examination team was interdisciplinary and consisted of a gastroenterologist and a surgeon that have clincal experience in diagnosing and treating pouchitis. Before study launch, all of the participating raters received training on the PDAI endoscopic features and the pouchoscopy standards used for the trial. The assessment was done with a video labeling tool that was specially created for the study.

### Video labelling tool and data preprocessing

To create a dataset that makes all relevant information accessible, a video labeling tool was developed that divided recorded pouchoscopy videos into still images, allowing a frame-by-frame assessment.

C++ was used as the coding language while the Qt-Toolkit (Qt Project, Espoo, Finnland) was utilized as development framework. The OpenCV library was the used for video related operations. The pouchoscopy images that were used for network 1 derived from different endoscopic systems, so the images had to be preprocessed to adjust the resolution. The black borders around the images were cropped using a python script so that only the relevant information remains. Detailed information on the preprocessing steps are given in the Appendix.

The data augmentation process was conducted using established methods from the PyTorch library, including rotation, flip, crop, colour jitter. The pixels were normalized via mean image subtraction. The entire dataset of annotated images was divided into three distinct subsets: training (60%), validation (20%) and test data (20%). The test dataset was withheld from both the training and validation processes, allowing for a comprehensive validation phase.

To minimize potential data leakage arising from the limited number of patients (*n* = 10), we grouped consecutive frames with identical labels. This process ensured that such sequences with related content were assigned to only one of the subsets. Splitting the data at patient level was not feasible in this pilot study.

### Network architecture

In consideration of the restricted size of the dataset, pre-trained CNNs were used as the backbone for our neural networks. This approach has the advantage of reducing the training effort, as pre-trained networks are capable of distinguishing fundamental image features, such as straight lines and edges.

An initial screening evaluated several candidates (ResNet50, ResNet101, ResNet152, ResNeXt50, ResNeXt101). Based on performance, ResNeXt101 was selected for network 1 and the quality assessment branch of network 2. ResNet152 was employed for the inflammation classification branch of network 2. Detailed results of the screening process and the hyperparameters are given in the Appendix.

**Fig. 1:**
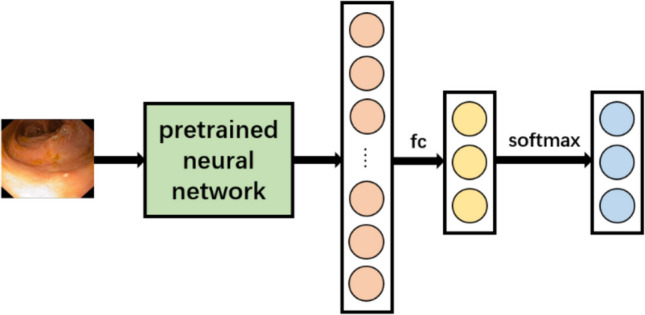
Architecture of network 1. fc, fully connected layer

Two distinct networks were developed. The objective of network 1 was to ascertain the presence of inflammation. It is a one-label classification task, with three potential outputs: ‘healthy’, ‘inflammation’ and ‘not assessable’. Figure 1 illustrates the data flow within the network.

Initially, the image is processed by a pre-trained network and then passed through a fully connection layer and a softmax activation function, resulting in the final outputs.

**Fig. 2:**
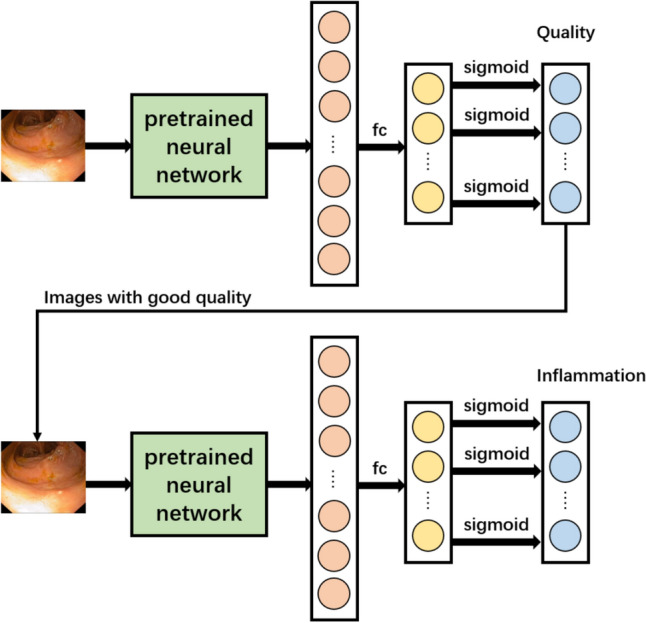
Architecture of Network 2. fc, fully connected layer

Network 2 was designed to classify endoscopic findings of pouchitis according to the PDAI score. This more complex, multi-label classification task necessitated an integrated solution. Figure 2 shows the architecture of the network, and it consits of two distinct subnetworks: one network for quality evaluation and one for inflammation classification. An image undergoes a quality assessment process, with images of satisfactory quality progressing to the subsequent network for inflammatory classification. If no inflammation is detected, the image is labelled as ‘healthy’. This approach limits the optional outputs of each subnetwork and was used to optimize the utilization of available resources.

### Validation and statistical evaluation

The final classification results were obtained from the previous excluded test set. This was done to ensure an unbiased evaluation. A tenfold cross-validation approach was applied to increase reliability. This allowed for the calculation of the mean performance on the test set, thereby increasing robustness and strengthening the validity of the results. Global values for accuracy, precision, sensitivity and specificity were computed for the individual classes and endoscopic markers. The F1 score was calculated to evaluate the overall performance of the CNN.

## Results

### Patient collective

The study comprised a total of ten videos, from which a total of 7130 images were extracted. After assessing all data, 2843 images were labelled ‘healthy’, 2326 images were labelled ‘not assessable’ and 1961 images were labelled ‘inflammation’. In the ‘inflammation’ class, a total of 531 images belonged to the endoscopic marker ‘oedema’, 816 images belonged to ‘granularity’, 1246 images belonged to ‘loss of vascular pattern’, 643 images belonged to ‘mucous exsudate’ and 1046 images belonged to ‘ulceration’.

### Labelling process

To avoid human bias, the labelling of the images was based on the four-eyes principle. This process involved two physicians, each meeting the minimum qualification of specialist certification, who possess substantial clinical experience in both the diagnosis and treatment of pouchitis. The examination team was interdisciplinary, consisting of a gastroenterologist and a surgeon.

### Network performance

The outcomes for the two networks are presented in Tables 1 and 2. Table 1 presents the results of network 1, which was designed to differentiate between the three primary classes: ‘healthy’, ‘not assessable’ and ‘inflammation’.

**Table 1:** Results of network 1 after tenfold cross-validation, values show the mean (standard deviation) of the result

Class	Accuracy	Precision	Sensitivity	Specificity	F1 score
Inflammation	85.28 (2.32)	73.79 (6.10)	71.78 (6.02)	90.35 (2.75)	72.52 (4.16)
Healthy	81.63 (2.15)	77.96 (5.70)	71.89 (5.13)	87.53 (3.64)	74.57 (3.21)
Not assessable	81.44 (1.42)	71.71 (4.49)	78.64 (3.55)	83.08 (3.04)	74.86 (2.21)
Average	82.78 (1.45)	74.49 (2.70)	74.10 (2.29)	86.99 (1.08)	73.98 (2.26)

**Table 2:** Results of network 2 after tenfold cross-validation, values show the mean (standard deviation) of the result

Class	Accuracy	Precision	Sensitivity	Specificity	F1 score
Oedema	93.61 (0.87)	34.17 (5.48)	44.35 (8.86)	95.98 (0.85)	37.91 (4.40)
Granularity	88.74 (2.23)	44.88 (8.23)	65.21 (11.14)	91.22 (3.07)	52.00 (6.49)
Friability	93.99 (1.27)	65.61 (10.87)	67.18 (7.98)	96.57 (1.21)	65.86 (8.05)
Loss of vascular pattern	91.38 (0.97)	70.09 (8.23)	61.36 (7.18)	95.94 (1.68)	64.73 (4.54)
Mucous exudate	92.22 (1.54)	25.29 (10.38)	52.89 (17.79)	93.88 (1.33)	33.29 (11.86)
Ulceration	87.53 (1.50)	46.93 (10.07)	38.00 (10.60)	94.12 (1.99)	41.21 (8.97)
Not assessable	77.69 (4.14)	55.85 (10.60)	71.29 (8.00)	74.07 (7.28)	59.01 (6.43)
Average	85.82 (8.99)	51.04 (14.86)	61.41 (17.04)	86.40 (13.32)	53.11 (13.77)

The results for each individual class, as well as the average performance across all classes per fold, were calculated. Accuracy ranged from 81.44% for the ‘not assessable’ class to 85.28% for the ‘inflammation’ class, with an overall average accuracy of 82.78%. Sensitivity showed greater variability, ranging from 71.78% for the ‘inflammation’ class to 78.64% for the ‘not assessable’ class. Specificity for the ‘inflammation’ class was 90.35%.

In terms of F1 scores, the ‘inflammation’ class had the lowest value at 72.52%, whereas the ‘not assessable’ class achieved the highest F1 score of 74.86%. The average F1 score across all classes was 73.98%.

The standard deviation of accuracy was relatively small, indicating consistent performance, while precision and sensitivity show more substantial fluctuations. The highest standard deviation (6.10%) occurred in precision for the ‘inflammation’ class, with sensitivity for the same class showing the second-highest standard deviation at 6.02%.

Table 2 presents the averaged results across the tenfold cross-validation of network 2, which was developed with the aim of providing a more detailed classification of the endoscopic markers associated with the ‘inflammation’ class. The overall averaged accuracy was 85.82%, with all individual classes achieving an accuracy greater than 70.00%. Within the ‘inflammation’ group, the lowest accuracy was observed for the ‘ulceration’ class (87.53%), while the ‘friability’ class exhibited the highest accuracy (93.99%). Precision values varied significantly, with the ‘mucous exudate’ class showing the lowest precision at 25.29% and the ‘loss of vascular pattern’ class achieving the highest precision at 70.09%. Sensitivity ranged from a low of 38.00% for the ‘ulceration’ class to a high of 67.18% for the ‘friability’ class. The ‘friability’ (65.86%) and ‘loss of vascular pattern’ (64.37%) classes outperformed others in terms of the F1 score within the ‘inflammation’ group. Regarding the standard deviation of the metrics, both sensitivity and specificity demonstrated substantial variability, whereas the deviations of other metrics were relatively smaller.

## Discussion

In recent years, AI has increasingly been integrated into medicine, with convolutional neural networks playing a central role. The number of AI-based programs in healthcare is steadily growing, with over 500 AI applications approved for patient care in the USA alone [19]. In the context of pouchitis diagnostics, this work serves as a pilot study, as no comparable studies have yet been published in this field.

Our findings align with those of other studies investigating other clinical topics. Sarwinda et al. [20] achieved comparable results for colorectal cancer detection using a ResNet-based image recognition program. However, their study was also a feasibility study with a small sample size and a restricted patient cohort. No comparable prospective studies using similar algorithms and larger patient populations were identified during the literature review.

The video labelling tool was designed to efficiently generate usable datasets in a user-friendly manner. It enabled and simplified the labelling of large datasets, was applied by multiple users without errors or confusion and fully met the requirements set out in the project design. The tool is also suitable for secondary use in other research projects. It is independent of the type of endoscopy, and the endoscopic features to be labelled are customizable, making it applicable to research beyond pouchitis diagnostics.

The findings of this study must be critically assessed from various perspectives. The PDAI score, introduced by Sandborn et al. in 1994, remains the gold standard in pouchitis diagnosis, although there have been studies that showed that none of the current scores provided acceptable results, and there is currently no sufficiently validated and reliable score for pouchitis diagnosis [21]. Ardalan et al. [22] proposed the Monash score, which incorporates only three endoscopic features: bleeding, erosions and ulcerations. Compared with the PDAI, the Monash score showed superior intra- and inter-rater reliability. However, the PDAI’s strength lies in its inclusion of clinical assessment, as both endoscopic features and histological biopsy analysis are secondary in our view. The results of network 1 support the hypothesis that the PDAI’s endoscopic features are only partially suitable for developing image recognition software. For a clinically deployable system, a validated, reliable pouchitis score is essential.

Larger datasets typically lead to better prediction outcomes for CNNs, so the dataset size often becomes a limiting factor in AI-based applications. This was also the case in this study. Additional pouchoscopy images from diverse sources would have improved network accuracy.

For optimal image recognition performance, dataset size is crucial, and future studies should include images from a broader range of pouchoscopies.

A low variability was found in the pouchoscopy images, probably due to the strict standardization of the pouchoscopy procedure. The resulting low variability may skew results positively, especially since the dataset was randomly split into training, validation and test sets, which could introduce overlap between consecutive images of the same pouchoscopy. On the other hand, a lack of standardized data collection can pose challenges for dataset quality. Data inconsistencies arise when multiple examiners from different centers collect data over extended periods. This is known as ‘distribution shift’ in deep learning, though this issue was negligible in our study as all data came from a single centre with consistent equipment. The low variability observed may also be attributed to the small number of pouchoscopies (*n* = 10).

A potential source of bias is data leakage arising from the frame-wise splitting approach, despite the aforementioned mitigation strategies (sequence-level partitioning and a withheld test set). Some residual dependence between frames cannot be ruled out.

Future studies should increase patient numbers to better reflect biological variability. Including multiple examiners would also account for individual examination styles and endoscopic techniques, again improving dataset variability and realism. Additionally multi-equipment datasets should be included to improve external validity.

Class imbalance in training data is a common issue in deep learning, particularly for classification tasks. CNNs tend to predict majority classes (more frequently represented) better than minority classes. This was evident in the study, where the feature ‘loss of vascular pattern’ (1246 labelled images) was predicted more accurately than ‘mucous exudate’ (643 labelled images). Future studies should increase the number of pouchoscopies to balance the dataset. Additionally, using a different pouchitis score, such as the Monash score (with higher intra-rater reliability), may help improve dataset balance, though further validation is needed before routine implementation.

Another limitation of this study is the absence of a direct benchmark against human expert performance. Although the dataset was labelled by two experienced physicians following standardized procedures, we did not formally compare network performance with independent human diagnostic accuracy. Such direct comparison would be essential to determine whether AI-based approaches can complement clinical decision making and would provide more context for interpreting the observed performance metrics. Future studies should therefore integrate systematic comparisons with expert endoscopists to more accurately evaluate the potential clinical utility of CNNs in pouchitis diagnostics.

Lastly, an integrated-network architecture for image classification was chosen. This design does not explicitly train the network to recognize the ‘healthy’ class, which is a diagnosis of exclusion, potentially reducing specificity. It is likely that training the network to specifically detect each main class, including its associated features, would yield better results. However, this would require extensive training and computational resources. The architecture used in our study was chosen as a compromise to save computational resources and avoid classification conflicts, acknowledging that this choice could limit network performance.

In conclusion, this feasibility study, despite its limitations, was able to demonstrate that artificial neural networks can be used to develop an image recognition program, capable of detecting endoscopic features of pouchitis with reasonable results. However, our results underline that AI performance in this context remains below human expert level. The present work should therefore be seen as a proof of concept and not as evidence that AI is ready for clinical use in the context of pouchitis diagnostics. Further research with larger, multicentre, multi-equipment datasets, direct comparison with expert endoscopists and a reliable pouchitis scoring system is required to achieve a significant improvement in the performance.

## Figures and Tables

**Fig. 3:**
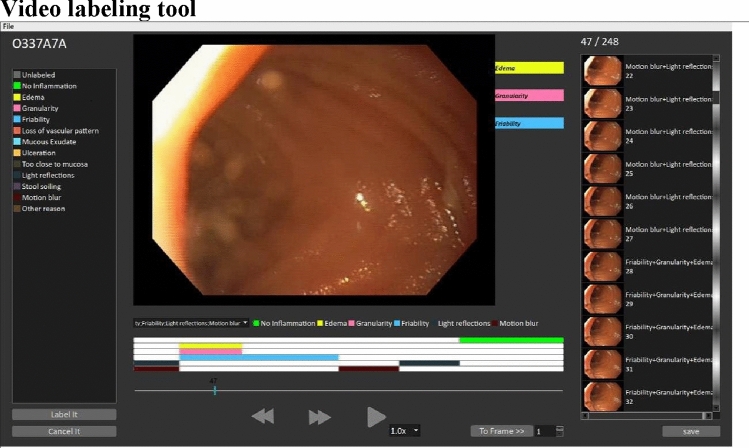
Videolabellingtool

## Data Availability

No datasets were generated or analysed during the current study.

## Appendix

See Fig. 3.

### Preprocessing of data

Since the images originate from different endoscopes the resolution is different (1920 × 1080, 1280 × 720, 768 × 576 pixels). The black borders around the images are cropped using a python script so that only the relevant information is remaining. Finally, the resulting images are resampled and stored.

To prepare the data for the training process, the entire dataset is split into training, validation and test set with the distribution of 60%, 20% and 20%, respectively. Since videos from ten patients were available, it is not possible to split the data at patient level. Therefore, to reduce data leakage, consecutive images with the same labels are grouped together. This ensures that image sequences with related content are not split up.

### Data augmentation

The data augmentation process was conducted using established methods from the PyTorch library, including random rotation, horizontal and vertical flip, affine transformations and colour jitter. The pixels were normalized via mean image subtraction.

### Network architecture and training

#### General setup

The SGD optimizer with momentum showed better performance than ADAM and was therefore used for all experiments.

To account for class imbalance, label weights are applied to the loss function according to the ratio of the amount of data.

#### Network 1

Figure 1 illustrates the architecture of the network to classify the three potential outputs: ‘healthy’, ‘inflammation’ and ‘not assessable’. To identify the most effective network architecture, we conducted an initial screening of several pre-trained convolutional neural networks, including ResNet50, ResNet101, ResNet152, ResNeXt50 and ResNeXt101. The hyperparameters were tuned using random search and a twofold cross-validation was utilized to reduce the computational cost. ResNeXt50 was chosen as most promising candidate. The optimized hyperparameters are presented in Table 3. The cross entropy loss function is used. The network is trained for 100 epochs.

**Table 3:** Hyperparameters for the training of network 1 using a pre-trained ResNext50

Initial learning rate	0.0001
Momentum	0.99
Batch size	64
Weight decay	0.01
Dropout rate	0.5

#### Network 2

Figure 2 illustrates the architecture of the network used to classify endoscopic findings of pouchitis according to the PDAI score. For network 2, the same approach was used as for network 1 to find the best overall architecture. To accomplish this, the two subnetworks for quality evaluation and inflammation classification were evaluated, one after the other. ResNeXt50 produced the best results for the quality evaluation network (hyperparameters in Table 4), whereas ResNet152 delivered the best performance for the inflammation classification network (hyperparameters in Table 5). The binary cross-entropy loss function is used. Both networks are trained for 100 epochs.

**Table 4:** Hyperparameters for the training of network 2 (quality evaluation part) using a pre-trained ResNext50

Initial learning rate	0.001
Momentum	0.9
Batch size	32
Weight decay	0.01
Dropout rate	0.4

**Table 5:** Hyperparameters for the training of network 2 (inflammation classification part) using a pre-trained ResNet152

Initial learning rate	0.001
Momentum	0.5
Batch size	16
Weight decay	0.01
Dropout rate	0.3
